# Cannabis and amphetamine use and its psychosocial correlates among school-going adolescents in Ghana

**DOI:** 10.1186/s13034-019-0293-0

**Published:** 2019-08-29

**Authors:** Kwaku Oppong Asante

**Affiliations:** 1 Department of Psychology, University of Ghana, P. O. Box LG 84, Legon, Accra, Ghana; 2 Department of Psychology, University of the Free State, Bloemfontein, South Africa; 3 Institute for Psychosocial Research on Child and Adolescent Wellbeing (IPRECAW), Accra, Ghana

**Keywords:** School-going adolescents, Amphetamine use, Cannabis use, Risk factors, Ghana

## Abstract

**Background:**

The aim of this study was to examine the prevalence of cannabis and amphetamine use and to determine its associated factors among school-going adolescents in Ghana.

**Method:**

The 2012 Ghanaian Global School-based Student Health Survey on 3632 adolescents aged 11–19 years (*mean* = 15.1 years; *SD* = 1.4) was used. Participants for this study were sampled from selected junior (JHS) and senior high schools (SHS) in all the 10 administrative regions of Ghana. A two-stage cluster sampling design was used to select 25 senior high schools to represent all the 10 regions of Ghana. Information was collected with a self-administered structured questionnaire that contained information on demographics, alcohol, tobacco and other drug use, violence, and a range of other health-related behaviours.

**Results:**

The result showed that past-month cannabis use was 5.3% and lifetime amphetamine use was 7.1% among students. In multivariate model, after controlling for other variables, school truancy and current cigarette smoking were associated with both past-month cannabis and lifetime amphetamine use. The number of close friends was associated with only past-month cannabis use. School environment factors (bullying victimisation and having been attacked) and parental substance use were associated with lifetime amphetamine use.

**Conclusion:**

This study identified a number of risk factors, including parental substance use and various risk behaviours, for both past-month cannabis and lifetime amphetamine use. School-based health intervention programmes should be developed taking into consideration the risk factors associated with cannabis and amphetamine use among school-going adolescents.

## Introduction

Illicit drug use contributes significantly to the global burden of disease, and thus is considered an emerging public health problem [1, 2]. According to the United Nations Office on Drugs and Crime (UNODC), the global prevalence of illicit drug use (including amphetamines, cannabis, cocaine, opioids, etc.) in 2015 was 5.3% [3]. The same report also indicated that cannabis, amphetamine-type stimulants, cocaine, and opioids were the most commonly used illicit drugs [3].

In a South African population-based survey conducted among individuals aged 12 years and older in 2012, past 3-month prevalence of illicit drug use was 4.4% [4]. A Ghanaian population-based national study conducted in 2008 among school-going adolescents, found the prevalence of past 1-month (any) drug use to be 3.6% [5]. Earlier follow-up studies among adolescents in Ghana reported prevalence rates of 2.6% and 7.2% for past-month cannabis use [6, 7].

Previous studies have established that specific sociodemographic factors are associated with both cannabis and amphetamine use, including male gender [5, 8] and older age [5, 6]. Furthermore, certain mental health-related behaviours such as anxiety [9, 10], loneliness [8], suicidal behaviour [11] and health risk behaviours including sexual risk behaviours [8, 12, 13], and current smoking [8, 14] have been found to be associated with amphetamine and cannabis use. The literature has shown interpersonal factors within the school environment to be related to amphetamine and cannabis use: being bullied [13, 15], physical fighting and being physically attacked [15], school truancy [14–16], lack of peer support [14], having a greater number of friends [17, 18] and hunger [8]. In addition, parental attributes such as parental substance use [9, 19], lack of parental support and monitoring [14], lack of parental connectedness [9] and lack of maternal demandingness [8] have been shown to influence amphetamine and/or cannabis use among school-going adolescents.

Within the Ghanaian context, no study has explored factors related to past-month cannabis and lifetime amphetamine use among school-going adolescents. Previous studies have predominantly focused on substance use (particularly tobacco and alcohol use) and its associated factors [6, 19, 20]. For example, Doku et al. [6] reported elevated levels of alcohol use and further indicated that alcohol use among school-going adolescents was associated with higher material affluence. Similarly, in their examination of the relationship between family dynamics and students’ alcohol use, Asiseh et al. [19] revealed that parental alcohol use increased the odds of the adolescents’ alcohol use irrespective of gender.

However, we are not well informed about the factors associated with cannabis and lifetime amphetamine use among school-going adolescents in Ghana. Additionally, since culture substantially influences human behaviour, the determinants of substance use as reported in developed Western countries (lack of parental support and monitoring, anxiety, loneliness, peer support, and sexual risk behaviours) [10, 14, 16] may not be applicable to high school students in Ghana.

In order to adapt interventions for illicit drug use among school-going adolescents in Ghana, national population-based prevalence data on cannabis and amphetamine use are needed. Therefore, the purpose of this secondary analysis is to estimate the prevalence of cannabis and amphetamine use and its associated factors among school-going adolescents using a nationally representative school-based survey conducted in 2012. This study focused on past-month cannabis use and lifetime amphetamine use because regular cannabis use is more common among this population than regular amphetamine use [1–3]. The findings of this study could inform interventions that target high school students who may be at risk for regular cannabis and lifetime amphetamine use.

## Methods

### Participants and procedure

Data for this study were obtained from the Ghana Global School-based Student Health Survey (GSHS) conducted in 2012 [21]. This survey was conducted through a partnership between the World Health Organization (WHO), the Center for Disease Control and Prevention (CDC), Middle Tennessee State University and the Ghana Education Service (GES). The data were collected using a cross-sectional survey design among WHO countries which were interested in examining the behavioural risk factors and protective factors in several domains of functioning among school-going adolescents. Data were collected through the use of close-ended structured questionnaires administered to the students. The GES’s policies on ethics regarding the use of students in survey studies were adhered to in the data collection. Written informed consent was obtained from students aged 18 years and above, while parental consent was taken for students who were less than 18 years prior to their participation in the study. As stipulated by GSHS, participation in the study was voluntary, anonymous, and confidential. The response rate was 74%.

### Sampling procedure

Participants were sampled from selected junior (JHS) and senior high schools (SHS) in all 10 administrative regions of Ghana. A two-stage cluster sampling design was used to select 25 senior high schools to represent all 10 regions of Ghana. Selection of schools at the first stage of sampling was based on a probability proportional to size of enrollment. At the second stage, a random sampling technique was used to select the classes in each school. This allowed every student to have an equal chance of being selected for the study. Numerical weights were applied to each student record to enable generalization of results to the eligible population. The students were relatively equally split across the four senior high school grade levels.

### Measures

The Ghana Global School-based Student Health Survey (GSHS) utilised a questionnaire that contained information on demographics, alcohol, tobacco, and other drug use, violence, and a range of other health-related behaviors [21, 22]. The Ghanaian version of the Global Student Health Survey was piloted and found to be culturally appropriate for use within Ghana [5]. The variables used in this study are described in Table 1.

**Table 1:** Independent variables derivation from survey data

Variable	Survey question	Original response options	Recoded
Age	How old are you?	11–18 years (coded categorically)	N/A
Sex	What is your sex	1 = male; 0 = female	N/A
Anxiety	During the past 12 months, how often have you been so worried about something that you could not sleep at night?	1 = never to 5 = always	1–3 = 0 and 4–5 = 1
Loneliness	During the past 12 months, how often have you felt lonely?	1 = never to 5 = always	1–3 = 0 and 4–5 = 1
Suicidal Ideation	During the past 12 months, did you ever seriously consider attempting suicide?	Yes = 1; no = 2	Yes = 1 and no = 0
Suicidal plan	During the past 12 months, did you make a plan about how you would attempt suicide?	Yes = 1; no = 2	Yes = 1 and no = 0
Suicidal attempt	During the past 12 months, how many times, did you actually attempted suicide	1 = 0 times to 5 = 6 times or more	1 = 0 and 2–5 = 1
School truancy	During the past 30 days, how many days did you miss classes or school without permission?	1 = 0 days to 5 = 10 or more days	1 = 0 and 2–5 = 1
Bullied	During the past 30 days, how many days were you bullied?	1 = 0 days to 7 = all 30 days	1–3 = 0 and 4–7 = 1
Physically attacked	During the past 12 months, how many times were you physically attack?	1 = 0 times to 8 = 12 or more times	1 = 0 and 2–8 = 1
In a physical fight	During the past 12 months, how many times were you in a physical fight?	1 = 0 times to 8 = 12 or more times	1 = 0 and 2–8 = 1
Hunger	During the past 30 days, how often did you go hungry because there was not enough food in your home?	1 = never to 5 = always	1–3 = 0 and 4–5 = 1
Sexual risk behaviour	During your life, with how many people have you ever had sexual intercourse	1 = never had intercourse to 7 = 6 or more people	1 = 0 and 2–7 = 1
Close friends	How many close friends do you have?	1 = 0 friends to 4 = 3 or more close friend	1 = 0 and 2–4 = 1
Peer support	During the past 30 days, how often were most of the students in your class kind and helpful?	1 = never to 5 = always	1–3 = 0 and 4–5 = 1
Current smoking of cigarette	During the past 30 days, how many days did you smoke cigarette?	1 = 0 days to 7 = all 30 days	1 = 0 and 2–7 = 1
Parental tobacco use	Which of your parents or guardian use any form of tobacco?	1 = never to 4 = both	1 = 0 and 2–4 = 1
Parental monitoring	During the past 30 days, how often did your parents or guardians check to see if your homework was done?	1 = never to 5 = always	1–3 = 0 and 4–5 = 1
Parental understanding	During the past 30 days, how often did your parents or guardians understand your problems and worries?	1 = never to 5 = always	1–3 = 0 and 4–5 = 1
Parental bonding	During the past 30 days, how often did your parents or guardians really know what you were doing you’re your free time?	1 = never to 5 = always	1–3 = 0 and 4–5 = 1
Parental intrusion of privacy	During the past 30 days, how often did your parents or guardians go through your things without your approval?	1 = never to 5 = always	1–3 = 0 and 4–5 = 1
Cannabis use	During the past 30 days, how many times have you used marijuana (also called weed, Jah, Indian hemp, ahabammmono, and ganja)?”	1 = 0 days to 5 = All 30 days	1 = 0 and 2–5 = 1
Amphetamine	During your life, how many times have you used amphetamine or methamphetamine (also called ice or yellow)	1 = 0 times to 5 = 20 or more times	1 = 0 and 2–5 = 1

### Data analysis

Sample weights were applied in all analyses to reduce bias from non-response, improve generalisability to the population, and further to reduce bias on the differing pattern of non-response. All variables were re-coded on a dichotomous scale as in other existing GSHS studies [11, 12, 19, 20]. The primary analyses were performed in two steps to determine factors most strongly associated with cannabis and lifetime amphetamine use in adolescents. First, bivariate analyses using the Chi-square (χ^2^) test were used to examine possible associations between the explanatory variables and past-month cannabis and lifetime amphetamine use. In the second step, Multinomial logistic regression analyses were conducted to examine the independent predictors of substance use. The results from the regression analyses are presented as odds ratios (OR) with 95% confidence intervals (CI). Statistical significance was defined as two-tailed *p*-value < 0.05 in all analyses. The Statistical Package for the Social Sciences (SPSS) version 23.0 was used to conduct data analyses.

## Results

### Sample characteristics

A total of 3632 school-going adolescents aged 11–19 (*mean* = 15.1 years; *SD* = 1.4) participated in the study. This sample included 1932 (53.2%) males and 1662 (45.8%) females. Gender data were missing for 38 participants (1%). About a third of the students, (n = 1062; 32.5%) were aged 18 years or older. Students aged 14 years and below comprised 24.4% of the sample, those aged 15 years constituted 13.8%, while those aged 16 years and 17 years constituted 12.2% and 16.9% of the sample respectively. Over half of the students (54.5%) were in senior high schools while the remaining 45.5% were in junior high school. Past-month cannabis use was 5.3% and lifetime amphetamine use was 7.1% among students.

### The relationship between cannabis and amphetamine use and their associated factors

The bivariate analysis of the factors associated with cannabis and amphetamine use among school-going adolescents in Ghana are presented in Table 2. Gender and age were not associated with either past-month cannabis use or lifetime amphetamine use. Mental health variables such as loneliness, and suicidal behaviour (i.e. ideation, plan, and attempt) were related to cannabis use but only loneliness, suicidal plan, and attempt were related to amphetamine use. Personal attributes such as truancy and cigarette smoking were independently associated with both past-month cannabis use and lifetime amphetamine use. A higher number of close friends was only associated with past-month cannabis use.

**Table 2:** Bivariate analysis of the factors associated with cannabis and amphetamine use among school-going adolescents in Ghana

Variables	Past-month cannabis use *N* = 184			Lifetime amphetamine use *N* = 238		
	No (%)	Yes (%)	*p*-value	No (%)	Yes (%)	*p*-value
Demographics						
Gender			0.540			0.450
Male	95.1	4.9		93.3	6.7	
Female	94.6	5.4		92.6	7.4	
Age in years			0.055			0.120
11–17 years	94.2	5.8		92.4	7.6	
18 years and above	95.8	4.2		93.9	6.1	
Mental health problems						
Anxiety			0.068			0.052
Yes	91.7	8.3		87.6	12.4	
No	95.3	4.7		93.8	6.2	
Loneliness			0.032			0.018
Yes	91.5	8.5		88.7	11.3	
No	95.4	4.6		93.7	6.3	
Suicidal ideation			0.011			0.091
Yes	87.9	12.1		85.5	14.5	
No	96.6	3.4		95.0	5.0	
Suicidal plan			0.019			0.049
Yes	90.3	9.7		88.0	12.0	
No	97.1	2.9		95.3	4.7	
Suicidal attempt			0.028			0.012
Yes	85.5	14.5		82.3	17.7	
No	97.8	2.2		96.3	3.7	
Personal attributes						
Truancy			< 0.001			< 0.001
Yes	89.0	11.0		87.6	12.4	
No	98.2	1.8		96.0	4.0	
Smoked cigarette			< 0.001			0.003
Yes	54.9	45.1		56.2	43.8	
No	97.3	2.7		95.5	4.5	
Close friends			0.021			0.610
Yes	94.9	5.1		92.9	7.1	
No	95.5	4.5		93.7	6.3	
Peer support			0.680			0.720
Yes	94.6	5.4		92.7	7.3	
No	94.9	5.1		93.0	7.0	
Sexual risk behaviour			0.061			0.065
Yes	88.0	12.0		86.1	13.9	
No	96.6	3.4		94.8	5.2	
School environmental factors						
Bullying victimization			0.044			< 0.001
Yes	91.7	8.3		89.3	10.7	
No	98.0	2.0		96.7	3.3	
Physically attacked			0.060			< 0.001
Yes	90.4	9.6		87.6	12.4	
No	97.8	2.2		96.5	3.5	
Physical fight			0.058			0.049
Yes	90.7	9.3		88.6	11.4	
No	97.1	2.9		95.3	4.7	
Hunger			0.030			0.080
Yes	90.9	9.1		88.6	11.4	
No	95.4	4.6		93.6	6.4	
Parental attributes						
Parental substance use			0.018			< 0.001
Yes	81.7	18.3		79.6	20.4	
No	96.5	3.5		94.8	5.2	
Parental monitoring			0.070			0.270
Yes	95.6	4.4		92.3	7.7	
No	94.3	5.7		93.3	6.7	
Parental understanding			0.010			0.620
Yes	96.1	3.9		92.7	7.3	
No	93.9	6.1		93.2	6.8	
Parental bonding			0.110			0.890
Yes	95.6	4.4		93.2	6.8	
No	94.3	5.7		93.0	7.0	
Parental intrusion of privacy			0.060			0.090
Yes	95.4	4.6		93.6	6.4	
No	93.9	6.1		92.1	7.9	

School environmental factors such as bullying victimisation and being physically attacked were associated with lifetime amphetamine use whilst bullying victimisation and hunger were related to past-month cannabis use. Parental substance use was related to both past-month cannabis use, and lifetime amphetamine use, but parental understanding of adolescents was only associated with past-month cannabis use.

### Predictors of cannabis and amphetamine use among school-going adolescents

The predictors of both past-month cannabis use, and lifetime amphetamine use are presented in Table 3. In multivariate analysis, after controlling for other variables, school truancy (OR = 3.34; 95% CI = 1.88–5.92; *p* < 0.001) and current smoking (OR = 12.48; 95% CI = 6.48–24.02; *p* < 0.001) were associated with past-month cannabis use. A greater number of close friends was positively associated with only past-month cannabis use (OR = 2.37; 95% CI = 1.19–4.71; *p* < 0.05). The results further showed in adjusted analysis that school truancy (OR = 1.74; 95% CI = 1.13–2.68; *p* < 0.05), current smoking (OR = 4.74; 95% CI = 2.50–9.00; *p* < 0.001), school environment factors such as bullying victimisation (OR = 2.09; 95% CI = 1.27–3.43; *p* < 0.01) and having been attacked (OR = 2.16; 95% CI = 1.36–3.45; *p* < 0.01), as well as parental substance use (OR = 2.45; 95% CI = 1.45–4.13; *p* < 0.01) were associated with lifetime amphetamine use.

**Table 3:** Association with cannabis and amphetamine use among school-going adolescents in Ghana

Variables	Past-month cannabis use		Lifetime amphetamine use	
	AOR	95% CI	AOR	95% CI
Demographics				
Age in years				
11–17 years	1	–	1	–
18 years and above	0.91	0.51–1.62	0.98	0.62–1.55
Sex (male)	0.74	0.43–1.28	1.07	
Mental health problems				
Anxiety	0.71	0.34–1.50	1.21	0.71–2.07
Loneliness	0.99	0.51–1.91	1.08	0.64–1.81
Suicidal ideation	1.84	0.97–3.50	1.51	0.88–2.60
Suicidal plan	1.43	0.75 –2.73	1.18	0.68–2.03
Suicidal attempt	1.40	0.70 –2.80	1.44	0.81–0.59
Personal attributes				
School truancy	3.34	1.88–5.92***	1.74	1.13–2.68*
Smoked cigarette	12.48	6.48–24.02***	4.74	2.50–9.00***
Close friends	2.37	1.20–4.71*	1.43	0.79–2.60
Peer support	1.50	0.83–2.72	1.31	0.83–2.07
Sexual risk behaviour	1.71	0.92–3.20	1.59	0.93–2.71
School environmental factors				
Bullying victimisation	1.65	0.87–3.13	2.09	1.27–3.43**
Physically attacked	1.61	0.89–2.93	2.16	1.36–3.45**
Physical fight	1.54	0.84–2.82	0.94	0.58–1.52
Hunger	0.99	0.49–1.99	1.35	0.78–2.33
Parental attributes				
Parental substance use	1.88	0.97–3.64	2.45	1.45–4.13**
Parental monitoring	0.90	0.50–1.79	1.31	0.82–2.11
Parental understanding	0.75	0.39–1.41	1.58	0.99–2.51
Parental bonding	0.91	0.48–1.74	1.07	0.67–1.73
Parental intrusion of privacy	1.04	0.60–1.80	1.09	0.71–1.68

*AOR* adjusted odds ratio for all factors which appear in table, *CI* confidence interval

*p < 0.05; ** *p *< 0.01; *** *p *< 0.001

## Discussion

The aim of this study was to examine the prevalence of past-month cannabis use and lifetime amphetamine use and to determine associated factors among school-going adolescents in Ghana. A prevalence rate of 5.3% and 7.1% were found for past-month cannabis use and lifetime amphetamine use respectively. The high prevalence of past-month cannabis use in this study is lower to the reported rate of 7.2% found among adolescents in 2012 [6] but seems to indicate an increase compared to an earlier study that reported a prevalence rate of 2.6% [7]. The prevalence rate of 5.3% reported in this study is similar to a UNODC report [1] indicating that adolescent past 30-day cannabis use was also low in Nigeria (4.4%) and Morocco (4.0%). A recent study also reported past 30-day cannabis use prevalence rates of 5.3%, 4.6% and 4.3% for Namibia, Swaziland, and Mauritius respectively [8]. In this study, the prevalence rate for lifetime amphetamine use was 7.1%, which is comparable to what has been reported among school-going adolescents in previous studies within sub-Saharan Africa [4, 9, 23]. The high prevalence rate for lifetime amphetamine use as found in this study is similar to a UNODC report which found a past year amphetamine prevalence rate of 7.6% among students in Ghana [2]. The current trend for cannabis use in the sub-Saharan Africa region is lower than the rates reported in this study.

The results further showed that age and gender were not significantly associated with either past-month cannabis use and lifetime amphetamine use. These results contradict previous studies that have established such associations [5, 8, 13]. Mental health variables such as anxiety, loneliness and suicidality did not predict either past-month cannabis or lifetime amphetamine use. These results contradict previous studies that have established such associations [8–11].

In this study, parental substance use was found to be associated with lifetime amphetamine use. This means that school-going adolescents who reported parental substance use were more likely to engage in substance use. This result confirms evidence from previous studies [9, 19] which indicate that parental engagement in a behavior is a huge predictor of offspring engaging in the same behaviour. Determining the association between parental substance use and adolescent substance use later in life is not straightforward [24]. This relationship between familial substance use and the likelihood of substance use in adolescence has been discussed through three (3) main pathways. One school of thought argues that family members with substance use problems may serve as behavioural models for young people living in the same household [19]. The second perspective indicates that family members living with problematic substance use may also store drugs and/or alcohol in the house making these substances more readily available to young people [25]. The third pathway is that substance use has a significant genetic component which explains why children of parents who use substances also use substances [26]. In Ghana, parental substance use may serve as a behavioural model which young people living in the same household imitate. Thus, their behaviour may send a message to these adolescents that it is acceptable for them to do the same.

The results further showed that school-related variables such as bullying victimisation and being physically attacked were associated with lifetime amphetamine use. The relationship between being physically attacked and lifetime amphetamine use is exceedingly complex and may be moderated by a host of individual and environmental factors. It is, however, possible that interpersonal level risk factors within the school environment may have played a role in this association, as indicated by previous studies [13, 15]. Additionally, the relationship between bullying victimisation and lifetime amphetamine use, could possibly be due to prior bullying victimisation that may predispose an adolescent to use amphetamine as a maladaptive coping strategy. This finding may also provide some support for the stress coping and self-medication model where recipients of peer victimisation, particularly those with poor coping strategies or self-regulatory processes may use substances as a way to deal with the pains associated with the victimisation experience [27–29]. With no anti-bullying policy in place within Ghanaian schools, in addition to the high prevalence of bullying reported among adolescents [30], this finding is a wake-up call for the Ghana Education Service (GES). The GES should consider these pathways in policy considerations for staff gatekeeping training programmes to address bullying victimisation.

Previous studies have reported that having more friends is protective against substance use [17, 18]. However, the inverse was found in the present study where the odds of cannabis use increased with a higher number of close friends. It has been reported that friendship provides a channel for adolescents to learn new social skills and subsequently experience positive developmental outcomes. However, it is possible that within the context of this study, having several friends led to the development of negative peer risk behaviours including substance use. Plausibly, such negative peer relationships may be associated with heightened health risk behaviours such as cannabis use, and thus underscore the need to emphasize supportive relationships between peers and develop strategies to promote positive peer support.

The findings also reveal that school truancy was a risk factor for both past-month cannabis use and lifetime amphetamine use. Consistent with the results of this study, several studies have established a relationship between school truancy and illicit substance use (i.e. cannabis and amphetamine use) [14–16, 19]. The relationship between truancy and substance use may be attributed to the weakened sense of school belonging among school-going adolescents. Schools are key social institutions which play an important role in constraining problem behaviours among adolescents [31]. However, since adolescence is also a time of increasing independence and searching for autonomy [32], reduced school engagement may also expose young people to health-compromising behaviours including substance use. It should be noted, though, that while truancy was associated with substance use, not all truants use substances [33].

Furthermore, school-going adolescents who smoked cigarettes were 12.5 and 4.8 times more likely to be past-month cannabis users and lifetime amphetamine users respectively. The presence of clustering of cigarette use with other illicit substance use including cannabis use has been reported in previous studies [8, 14, 34]. These findings underscore the need for the development of poly-drug use interventions among school-going adolescents.

### Limitations of the study

This study has some limitations. Firstly, the key outcome variables, amphetamine and cannabis use, were self-reported. Self-report may be confounded by systematic and social desirability biases. Secondly, the majority of the measures used were single item measures which only allows narrow assessment of these variables. Thirdly, results are based on a cross-sectional database, thus limiting our ability to establish causality. Longitudinal studies concerning amphetamine and cannabis use among school-going adolescents are needed. Finally, the study only included adolescents attending school; out of school, 11–18 year-olds were not included. Therefore, findings are not representative of all adolescents in this age group. Despite these limitations, this is one of the first cross-sectional studies to have used nationally representative data to explore the prevalence of cannabis and amphetamine use and their associated factors among school-going adolescents.

## Conclusion

This study was conducted to examine the prevalence and factors associated with amphetamine and cannabis use among school-going adolescents in Ghana. Although the prevalence of amphetamine use in this adolescent population in Ghana may not be as high as in some high-income countries, this study found a high prevalence of cannabis use. This study identified a number of risk factors for both cannabis and amphetamine use including truancy and cigarette smoking. While parental substance use, bullying victimisation and having been physically attacked were risk factors for amphetamine use, having a greater number of close friends was a risk factor for cannabis use. School-based health programmes should be developed which take the risk factors associated with cannabis and amphetamine use among school-going adolescents in Ghana into consideration.

## Data Availability

Data is freely available online from Ghana Global School-based Student Health Survey (GSHS).
